# Hiss and snort call types of wild-living giraffes *Giraffa camelopardalis*: acoustic structure and context

**DOI:** 10.1186/s13104-017-3103-x

**Published:** 2018-01-09

**Authors:** Elena V. Volodina, Ilya A. Volodin, Elena V. Chelysheva, Roland Frey

**Affiliations:** 1 Scientific Research Department, Moscow Zoo, Moscow, 123242 Russia; 2 Department of Vertebrate Zoology, Faculty of Biology, Lomonosov Moscow State University, Moscow, 119234 Russia; 3 Mara-Meru Cheetah Project, Sarit Centre, P.O. Box 1611, Nairobi, 00606 Kenya; 4 Department of Reproduction Management, Leibniz Institute for Zoo and Wildlife Research, 10315 Berlin, Germany

**Keywords:** Mammal, Acoustic communication, Vocalization, Emotional arousal, Giraffe, Hiss, Snort, Ungulate, Vigilance behaviour, Ruminant, *Giraffa camelopardalis*

## Abstract

**Objectives:**

Vocalization as part of vigilance behaviour is widespread across animal taxa, including ruminants. Calls of wild-living giraffes have never been recorded and spectrographically investigated. This study reports the acoustic structure of vigilance-related hiss and snort calls of wild-living giraffes *Giraffa camelopardalis*.

**Results:**

The hiss and snort calls were emitted during five recording sessions produced by nine individual giraffes (8 adults and 1 subadult) in their natural environment in Namibia (3 individuals) and Kenya (6 individuals). These calls attended vigilance behaviour toward humans in hides or in vehicles and cheetahs as natural predators of giraffe young. This study provides spectrographic analyses of 22 hiss and 20 snort calls. The giraffe hisses were broadband vocalizations of an average duration of 0.72 s (from 0.24 to 1.04 s) and a peak frequency of 0.69 kHz. The giraffe snorts were broadband pulsed calls of an average duration of 0.28 s (from 0.13 to 0.55 s), a peak frequency at 0.20 kHz and comprised a prominent low-frequency pulsation of 23.7 pulses/s. The acoustic structure of giraffe hisses is reminiscent of vigilance-related hisses of musk deer *Moschus moschiferus*. Giraffe snorts differ from snorts of other ruminants by their prominent pulsed pattern.

**Electronic supplementary material:**

The online version of this article (10.1186/s13104-017-3103-x) contains supplementary material, which is available to authorized users.

## Introduction

Vocalizations attend vigilance behaviour in many animal taxa [1]. The acoustic structure of these vocalizations may encode behavioural context and type of predator [2–5] as well as the degree of negative emotional arousal of the caller [6, 7] and thus the threat urgency [8–10]. These acoustic cues to the degree of urgency and arousal can be used for avoiding danger by conspecifics [8, 11, 12] or heterospecifics [13, 14].

In mammals, vigilance-related vocalizations are best studied for group-living rodents [15, 16], primates [5, 17] and carnivores [18–20], and have been reported for fourteen species of ruminants [21–42], including giraffe *Giraffa camelopardalis* [38–42]. Among reported audible [38–40] and infrasonic (14 Hz) vocalizations [41, 42], only presence of audible call types (snort, burst, grunt and humm) was confirmed by recent studies of captive giraffes [43, 44].

Giraffe hisses were verbally reported as nasal calls emitted in the context of approach investigation [38]. Giraffe snorts were verbally reported as short plosive grunts, being produced through the widened nostrils while the animal was standing and scanning one particular spot or direction as a reaction to a potentially threatening irritation [45–47] or immediately after a dominant giraffe bull had chased off an inferior bull [47]. Giraffe snorts were reported as an illustrative spectrogram and a wave-file based on recordings made in captivity using automate recording systems [43]; acoustic analyses of these calls were not provided. Calls of wild-living giraffes have never been recorded previously. The purpose of this study was to present the acoustic structure of the hiss and snort calls of wild-living giraffes.

## Main text

### Methods

Giraffe calls were collected in two natural localities: in February 2016 at the 15,000-hectare Okambara Ranch located 130 km east of Windhoek (Namibia) and in March and November–December 2016 in Maasai-Mara National Reserve and the adjacent area Mara Conservancy area (Kenya). During five recording sessions (2 in Namibia and 3 in Kenya, all conducted on different days), calls of 9 giraffes were recorded (3 individuals *G.c. giraffa* in Namibia and 6 individuals *G.c. tippelskirchi* in Kenya [48]), including 2 adult males, 2 adult females, 4 adults of unspecified sex and 1 subadult male.

For acoustic recordings (48 kHz, 16 bit, frequency range 20–20,000 Hz) we used Marantz PMD-660 or PMD-661 solid state recorders (D&M Professional, Kanagawa, Japan) with Sennheiser K6-ME66 or ME67 microphones (Sennheiser electronic, Wedemark, Germany). During recording, each call was labeled by the researcher’s voice to identify the target vocalizations against background noises, the behavioural context and (where possible), the individual identity, sex and age of a caller. All calls were produced spontaneously by the giraffes; researchers did not provoke the animals to vocalize.

At the Namibia locality calls were recorded from hides during daytime and at twilight. One hide was a potential feeding tree of giraffes with overhanging branches that covered the researcher thus preventing his visual detection by the giraffes. Another hide consisted of a row of dense bushes, approached by giraffes at twilight in the evening when moving to a nearby water place for drinking. The researcher sitting in this hide was partly visible to the giraffes at a distance of approximately 20 m.

In the Kenya locality, where leaving vehicles is prohibited, calls were recorded from a standing car during daytime at distance of 10–100 m to the giraffes. Two recordings were done when cheetahs *Acinonyx jubatus*, occasional predators of giraffe young [49, 50], were at 30–100 m to the target giraffes. The third recording was done on giraffe vigilance behaviour toward a standing car, in the absence of predators.

**Fig. 1:**
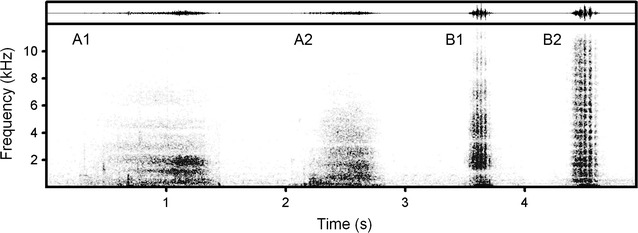
Two vigilance-related call types in the giraffe *Giraffa camelopardalis*. The spectrogram (below) and waveform (above) illustrate (**A1**) hiss of an adult giraffe of unspecified sex in Namibia; **A2** hiss of a subadult male giraffe in Kenya; **B1** snort of an adult giraffe of unspecified sex in Namibia; **B2** snort of an adult female in Kenya. In the waveform of the snorts, the low-frequency pulsation is visible as amplitude peaks. The spectrogram was created with 24 kHz sampling frequency, Hamming window, FFT 1024 points, frame 50% and overlap 93.75%. Original wav-files are available in the electronic supporting information (Additional file 1: Audio S1)

Calls of good quality were selected for acoustic analyses (24 kHz sampling frequency, Hamming window, FFT 1024 points, frame 50%, overlap 93.75%) using Avisoft SASLab Pro software (Avisoft Bioacoustics, Berlin, Germany). Based on the acoustic structure (Fig. 1) and the sounding (Additional file 1: Audio S1), we classified the calls into hiss and snort call types. For each call, we measured call duration from the screen with the standard marker cursor in the spectrogram window. After high-pass filtration (100 Hz, Gauss filter) we measured the maximum amplitude frequency (fpeak) and three quartiles (q25, q50 and q75), covering 25, 50 and 75% of call energy from the mean power spectrum of each call. For the snorts (all of them displayed low-frequency pulsation), we measured the pulse rate with the standard marker cursor. All measurements were exported to Microsoft Excel (Microsoft Corp., Redmond, WA, USA) for analyses. In total, we measured 22 hiss calls (21 from Namibia and one from Kenya) and 20 snort calls (1 from Namibia and 19 from Kenya), from 1 to 11 (mean ± SD = 4.7 ± 3.9) calls per individual.

Statistical analyses were made with STATISTICA, v. 8.0 (StatSoft, Tulsa, OK, USA); all means are given as mean ± SD, and differences were considered significant whenever *p* < 0.05. Nine of ten distributions did not depart from normality (Kolmogorov–Smirnov test, *p* > 0.05), so, we could use Student *t* test with Bonferroni correction for multiple comparisons to compare the parameter values between the hiss and snort calls.

### Results

Giraffe hisses were broadband vocalizations of duration 0.24–1.04 s and the peak frequency at 0.69 ± 0.61 kHz (Fig. 1, Table 1, Additional file 2: Table S1). Giraffe snorts were broadband vocalizations of duration 0.13–0.55 s and the peak frequency at 0.20 ± 0.29 kHz, displaying a prominent low-frequency pulsation ranging in rate from 18.3 to 32.8 pulses/s between calls (Fig. 1, Table 1). Compared to hisses, snorts were shorter, their peak frequency was lower, and the lower, medium and upper power quartiles were all higher (Table 1).

**Table 1:** Values (mean ± SD) of acoustic variables measured for hiss and snort call types of giraffe and Student *t* test results of their comparison

Call types	Duration (s)	fpeak (kHz)	q25 (kHz)	q50 (kHz)	q75 (kHz)	Pulse rate (Hz)
Hiss (*n* = 22)	0.72 ± 0.22	0.69 ± 0.61	0.71 ± 0.24	1.55 ± 0.37	2.94 ± 0.60	Non-pulsed
Snort (*n* = 20)	0.28 ± 0.10	0.20 ± 0.29	1.05 ± 0.47	2.23 ± 0.90	4.39 ± 1.64	23.7 ± 4.2
Student *t* test results	*t* = 8.34; ***p*** ***<*** ***0.001***	*t* = 3.11; ***p*** ***=*** ***0.003***	*t* = 3.03; ***p*** ***=*** ***0.004***	*t* = 3.21; ***p*** ***=*** ***0.003***	*t* = 3.84; ***p*** ***<*** ***0.001***	Not applicable

Designations: duration—call duration; fpeak—peak frequency; q25, q50 q75—lower, medium and upper quartiles. *p* estimates less than 0.01 (after Bonferroni correction) are shown in bolditalics

We observed that snorts were produced nasally by a sudden burst of air released from the nostrils. However, we could not establish whether the hisses were produced through the nose or via the opened mouth. Emission of hisses toward a hidden researcher was sometimes preceded by neck-shaking. Emissions of snorts were not preceded or accompanied by any prominent movements; only one adult female nodded during the emission of snorts. Hisses and snorts were either produced singly or in series lasting more than half an hour. Hisses and snorts could occur in the same call series. Calls within series were separated by time intervals of up to a few minutes.

Most hisses (20 of 22) were produced toward a researcher, hidden under the overhanging tree branches. At the day of the recording, this hide was approached by a group of 7 giraffes for feeding. The giraffes came so close that their heads and parts of their bodies were 3–5 m above the researcher. Only then the animals detected the researcher and started the emission of hisses. The researcher could see the giraffe heads through the branches against the bright sky, whereas the giraffes, despite their acute vision [51], could hardly see the researcher when looking from bright sunlight towards the dense foliage. Probably, the hissing was triggered by the sudden detection of human smell just underneath their food source, as the olfactory abilities of giraffes are good [52]. Despite hampered vision of the researcher at least two callers could be identified because calls arrived from different sides at the researcher. After some time, five giraffes went off, whereas two animals stayed at a distance of 8 m and one of them continued hissing. The caller passed a few steps from one side to the other in front of the hiding tree and then hissed. Before hissing, the caller often displayed neck-shaking.

Most giraffe snorts (19 of 20) were recorded, when the callers were in 30–100 m of female cheetahs with cubs. Cheetahs were active, either eating a kill or moving along groups of giraffes but did not pay evident attention to the giraffes. During one recording, an adult giraffe from a group of three animals spotted a group of three cheetahs (a mother with two cubs) eating a kill and started approaching them slowly. The giraffe produced three snorts, the first snort at a distance of 50–60 m, the second at 45–50 m and the third at more than 100 m, while retreating from the cheetahs. Intervals between the snorts were about 1 min. During another recording, a single subadult male produced a long series of snorts and one hiss after an adult cheetah female had passed by and the giraffe ran off for a few meters and then stopped, looking at her.

### Discussion

This study is the first describing the acoustic variables of calls of wild-living giraffes. Both the hiss and snort call types were rather soft and probably communicated vigilance at short range, as short-range ultrasonic alarm calls of ground squirrels [53, 54]. Whereas most hisses were recorded in Namibia and most snorts in Kenya, this discrepancy was probably related to the situation of higher potential danger and less predictability for giraffe callers in Namibia (where giraffes are legally hunted from the hides) than in Kenya (where the hunting is prohibited) and not to locality per se. Otherwise, the single hiss that was recorded in Kenya, was produced by a subadult male toward the cheetahs, that predate young giraffes [49, 50]. Thus, hisses prevailed in the contexts of higher tenseness/unexpectedness for a caller whereas the snorts prevailed in the contexts of less unexpectedness for a caller. Two different call types in response to different levels of threat are known for many species of mammals and birds [55].

We took one single captive giraffe snort from the supplementary material of the study of Baotic et al. [43] and measured its acoustic characteristics. The acoustics of this snort (duration = 0.21 s; fpeak = 0.09 kHz; q25 = 1.05 kHz; q50 = 1.68 kHz; q75 = 3.09 kHz; pulse rate = 26.5 Hz) were similar to those of the snorts measured in the current study (Table 1).

**Fig. 2:**
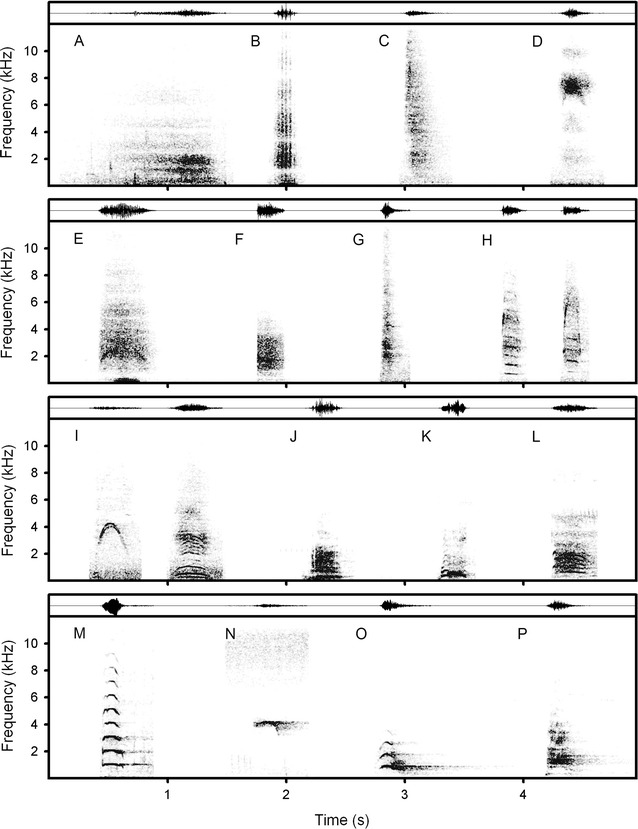
Vigilance-related calls across Ruminantia. **A** Hiss and **B** snort of a giraffe; **C** hiss of a musk deer *Moschus moschiferus*; **D** snort of a female goitred gazelle *Gazella subgutturosa*; **E** snort of a waterbuck *Kobus ellipsiprymnus*; **F** snort of a male impala *Aepyceros melampus*; **G** snort of a male Western tur *Capra caucasica cylindricornis*; **H** two snorts of a male klipspringer *Oreotragus oreotragus*; **I** two snorts of a male springbok *Antidorcas marsupialis*; **J** bark of a female greater kudu *Tragelaphus strepsiceros*; **K** bark of a white-tailed gnu *Connochaetes gnou*; **L** bark of a male Indian muntjac *Muntiacus vaginalis*; **M** bark of a female sambar deer *Rusa unicolor*; **N** bark of a female sika deer *Cervus nippon*; **O** bark of a female Siberian red deer *Cervus elaphus sibiricus*; **P** bark of a female Bactrian red deer *Cervus elaphus bactrianus*. The illustrative spectrograms are based on calls recorded from adult wild-living animals (except the Western tur, recorded in captivity) that vocalized at the sudden appearance of a human. During their vocalizations, the callers did not flee but either froze or slowly passed by the human. Spectrograms were created with 24 kHz sampling frequency, Hamming window, Fast Fourier Transform (FFT) 1024 points, frame 50% and overlap 93.75%. Original wav-files are available in the electronic supporting information (Additional file 3: Audio S2)

Giraffe nasal hisses and snorts can be compared by their acoustic structure with vigilance-related nasal hisses and snorts of other ruminants (Fig. 2, Additional file 4: Table S2). Giraffe hisses were the longest calls among ruminants, either nasal or oral (Additional file 4: Table S2). Probably this was the effect of the non-explosive air expulsion during the hisses and/or the effect of large body size (lung volumes) of the giraffe [56]. Similarly non-explosive air expulsion occurs only during the hisses of the much smaller-sized musk deer *Moschus moschiferus* (Fig. 2). Giraffe snorts had the lowest peak frequency among the snorts of other ruminants (Additional file 4: Table S2). This might also result from the giraffe’s large body size [56] and its respectively large vocal tract or larynx [57, 58].

Giraffes share the nasal vocal emission with goitred gazelle *Gazella subgutturosa* [35], impala *Aepyceros melampus*, Western tur *Capra caucasica cylindricornis*, klipspringer *Oreotragus oreotragus* and springbok *Antidorcas marsupialis* (Additional file 4: Table S2). These nasal calls are probably produced aerodynamically by vortices at the glottis or vocal tract narrowings [59–62], distinctive to oral calls (barks) of, e.g. white-tailed gnu *Connochaetes gnou*, sambar deer *Rusa unicolor* and sika deer *Cervus nippon* (Fig. 2, Additional file 4: Table S2) displaying fundamental frequency produced evidently by the vocal folds [63, 64]. In the springbok and klipspringer, superimposed fundamental frequency and aerodynamic whistle (Fig. 2, see also [36]) suggest biphonation, described for some cervids [24, 62, 65]. In the giraffe, the fundamental frequency was only reported for the humm vocalization [43], described based on automated recordings in the absence of researchers, so nasal or oral vocal emission and context of this vocalization could not be determined.

## Limitations

This pilot study had a few limitations:

- The study was conducted in one locality in Namibia and one in Kenya, what limits expansion of results for entire local populations of giraffes.
- Context of vocalizing could not be predicted and standardized, as giraffes vocalize very rarely. Two months of stay in Namibia (three researchers, 4–8 h of observations/day) and 1 year of stay in Kenya (one researcher, 8 h of observations/day) provided only five recording sessions.
- Only 5 of the 9 callers were sexed as vision was complicated by the foliage or twilight during observations in Namibia.

## Additional files

**Additional file 1: Audio S1.** Hisses and snorts of giraffes emitted toward potential threat in Namibia and Kenya. Hiss of an adult giraffe of unspecified sex from Namibia; hiss of a subadult male giraffe from Kenya; snort of an adult giraffe of unspecified sex from Namibia; snort of an adult female giraffe from Kenya.

**Additional file 2: Table S1.** Table with acoustic measurements of giraffe hisses and snorts for describing the acoustic features of these two call types.

**Additional file 3: Audio S2.** Hiss and snort of a giraffe; hiss of a musk deer; snort of a female goitred gazelle; snort of a waterbuck; snort of a male impala; snort of a male Western tur; two snorts of a male klipspringer; two snorts of a male springbok; bark of a female greater kudu; bark of a white-tailed gnu; bark of a male Indian muntjac; bark of a female sambar deer; bark of a female sika deer; bark of a female Siberian red deer; bark of a female Bactrian red deer.

**Additional file 4: Table S2.** Indicating call type, nasal/oral vocal emission and acoustic characteristics of vigilance-related vocalizations across Ruminantia.

## Abbreviations

fpeak: call maximum amplitude frequency
q25: call lower power quartile
q50: call medium power quartile
q75: call upper power quartile
