# Supplementary material for: Hiss and snort call types of wild-living giraffes Giraffa camelopardalis: acoustic structure and context
Source: BMC Res Notes. 2018 Jan 9;11:12. doi: 10.1186/s13104-017-3103-x (PMC5761111; doi:10.1186/s13104-017-3103-x)
Supplement: Supplementary file 4 — Additional file 4: Table S2. Indicating call type, nasal/oral vocal emission and acoustic characteristics of vigilance-related vocalizations across Ruminantia. [file 13104_2017_3103_MOESM4_ESM.pdf]

Supplementary Table

Call type, nasal/oral vocal emission and acoustic characteristics of vigilance-related vocalizations across Ruminantia

Species name	Latin name	Call type	Nasal/oral production	duration (s)	fpeak (kHz)	f0max (kHz)	Illustration
giraffe	*Giraffa camelopardalis*	hiss [*]	nasal [38]	0.72 [*]	0.69 [*]		Fig 1A, 2A
giraffe	*Giraffa camelopardalis*	snort [*]	nasal [**]	0.28 [*]	0.20 [*]		Fig 1B, 2B
musk deer	*Moschus moschiferus*	hiss [31,**]	not indicated	0.25 [31]	3.0 – 5.0 [31]		Fig 2C
goitred gazelle	*Gazella subgutturosa*	snort [35]	nasal [35]	0.18 [35]	3.06 [35]		Fig 2D
waterbuck	*Kobus ellipsiprymnus*	snort [**]	not indicated				Fig 2E
impala	*Aepyceros melampus*	snort [**]	nasal [**]				Fig 2F
Western tur	*Capra caucasica cylindricornis*	snort [**]	nasal [**]				Fig 2G
klipspringer	*Oreotragus oreotragus*	snort [36,**]	nasal [**]				Fig 2H
springbok	*Antidorcas marsupialis*	snort [**]	nasal [**]				Fig 2I
topi	*Damaliscus lunatus*	snort [34]	not indicated	0.22 [34]	2.03 [34]		
greater kudu	*Tragelaphus strepsiceros*	bark [**]	oral [**]				Fig 2J
white-tailed gnu	*Connochaetes gnou*	bark [**]	oral [**]				Fig 2K
four-horned antelope	*Tetracerus quadricornis*	bark [37]	not indicated				

Indian muntjac	*Muntiacus vaginalis*	bark [28,29]	oral [29]	0.24 [28]; 0.26 [29]	0.89 [28]	0.59 [29]; 0.66 [28]	Fig 2L
Chinese muntjac	*Muntiacus reevesi*	bark [30]	not indicated	0.27 – 0.51 [30]	0.67 – 1.12 [30]		
sambar deer	*Rusa unicolor*	bark [28]	oral [28]	0.15 [28]	1.61 [28]	0.98 [28]	Fig 2M
sika deer	*Cervus nippon*	bark [23,25]	oral [25]	0.12 [25]; 0.17 [23]		2.60 [23]; 2.67 [25]	Fig 2N
Siberian red deer	*Cervus elaphus sibiricus*	bark [**]	oral [**]	0.20 [24]		0.93 [24]	Fig 2O
Bactrian red deer	*Cervus elaphus bactrianus*	bark [**]	oral [**]				Fig 2P
Scottish red deer	*Cervus elaphus scoticus*	bark [23]	not indicated	0.22 [23]		0.15 [23]	
Canadian wapiti	*Cervus canadensis*	bark [21,22]	oral [21]	0.25 [21]			
European roe deer	*Capreolus capreolus*	bark [32,33]	not indicated		1.4-1.7 [32]		
Siberian roe deer	*Capreolus pygargus*	bark [32]	not indicated		1.0-1.4 [32]		
white-tailed deer	*Odocoileus virginianus*	snort [26,27]	oral [26]; nasal [27]	0.14 [26]; 0.25 [27]			

* this study.

** unpublished observations of the authors.
